# Bioinformatics combined with machine learning unravels differences among environmental, seafood, and clinical isolates of *Vibrio parahaemolyticus*

**DOI:** 10.3389/fmicb.2025.1549260

**Published:** 2025-03-19

**Authors:** Shuyi Feng, Padmini Ramachandran, Ryan A. Blaustein, Abani K. Pradhan

**Affiliations:** ^1^Department of Nutrition and Food Science, University of Maryland, College Park, MD, United States; ^2^Human Foods Program U.S. Food and Drug Administration, College Park, MD, United States; ^3^Center for Food Safety and Security Systems, University of Maryland, College Park, MD, United States

**Keywords:** comparative genomics, machine learning, *Vibrio parahaemolyticus*, virulence, antibiotic resistance

## Abstract

*Vibrio parahaemolyticus* is the leading cause of illnesses and outbreaks linked to seafood consumption across the globe. Understanding how this pathogen may be adapted to persist along the farm-to-table supply chain has applications for addressing food safety. This study utilized machine learning to develop robust models classifying genomic diversity of *V. parahaemolyticus* that was isolated from environmental (*n* = 176), seafood (*n* = 975), and clinical (*n* = 865) sample origins. We constructed a pangenome of the respective genome assemblies and employed random forest algorithm to develop predictive models to identify gene clusters encoding metabolism, virulence, and antibiotic resistance that were associated with isolate source type. Comparison of genomes of all seafood-clinical isolates showed high balanced accuracy (≥0.80) and Area Under the Receiver Operating Characteristics curve (≥0.87) for all of these functional features. Major virulence factors including *tdh*, *trh*, type III secretion system-related genes, and four alpha-hemolysin genes (*hlyA*, *hlyB*, *hlyC*, and *hlyD*) were identified as important differentiating factors in our seafood-clinical virulence model, underscoring the need for further investigation. Significant patterns for AMR genes differing among seafood and clinical samples were revealed from our model and genes conferring to tetracycline, elfamycin, and multidrug (phenicol antibiotic, diaminopyrimidine antibiotic, and fluoroquinolone antibiotic) resistance were identified as the top three key variables. These findings provide crucial insights into the development of effective surveillance and management strategies to address the public health threats associated with *V. parahaemolyticus*.

## 1 Introduction

*Vibrio parahaemolyticus* is a Gram-negative, halophilic bacterium that is widely distributed in estuarine, marine, and coastal surroundings, and frequently detected in diverse seafood products such as clams, shrimps, crabs, and oysters (Su and Liu, 2007). *V. parahaemolyticus* is an important foodborne pathogen that is responsible for illnesses associated with seafood throughout the world and is often linked to the consumption of raw or improperly handled seafood (DePaola et al., 2003). Typical signs and symptoms triggered by the infection of *V. parahaemolyticus* encompass watery diarrhea, abdominal cramps, nausea, vomiting, fever, headache, and bloody diarrhea (Centers for Disease Control and Prevention, 2013). Immunocompromised persons are at highest risk for morbidity and mortality (Centers for Disease Control and Prevention, 2013). Outbreaks/infections caused by *V. parahaemolyticus* usually happen in regions with high water temperatures. However, the ongoing climate changes are believed to expand the prevalence of *V. parahaemolyticus* geographically and increase human exposure to *V. parahaemolyticus* on a global scale (Zhang W. et al., 2023). Therefore, the development of efficient management strategies to control the spread of *V. parahaemolyticus* and minimize related food safety risks is needed.

Native to estuarine environments, *V. parahaemolyticus* can become a problematic contaminant among the microflora in shellfish as it takes on a broad niche range. In general, bacterial attachment and internationalization are described as the two critical processes mediating its transmission to and persistence in raw seafood (Brauge et al., 2024). Human consumption of contaminated seafood products may then result in the development of foodborne illness. However, the specific mechanisms involved in *V. parahaemolyticus* transmission and survival across diverse lifestyles, from the environment to seafood and consumers remain unclear. Thermostable direct haemolysin (TDH) and thermostable-related haemolysin (TRH) are the two major virulence factors in *V. parahaemolyticus* that may play important roles, as *tdh* and *trh* have been identified as reliable gene markers for the detection of pathogenic strains due to their prevalence in clinical isolates (Raghunath, 2015). Nevertheless, several studies have demonstrated that *tdh* and *trh* negative strains also cause infection, which indicates that additional virulence factors may be involved as well (Chao et al., 2010; Velazquez-Roman et al., 2012; Zha et al., 2023). Furthermore, while antibiotics have been widely adopted as the major treatment for *V. parahaemolyticus* infection, especially for severe cases (Loo et al., 2020), there is a growing concern for the emergence of antibiotic resistance among the species (Letchumanan et al., 2015; Letchumanan et al., 2016; Loo et al., 2020). Comparing the metabolism, virulence, and antibiotic resistance profiles of different *V. parahaemolyticus* isolates representing alternative lifestyles (i.e., waterborne, food-associated, and clinical) may provide a better understanding of its mechanisms for contamination, pathogenicity, and overall health risk.

Whole genome sequencing technologies have become increasingly utilized in the food industry for food safety monitoring assessment (Brown et al., 2019; Unrath et al., 2021). Given the complexity of sequencing data, machine learning (ML) can be applied to capture patterns in datasets with large quantities, and make robust predictions based on identified patterns (Tanui et al., 2022b; Karanth et al., 2022; Benefo et al., 2024a; Feng et al., 2024). Machine learning, particularly supervised ML, has demonstrated great applications in food safety such as predicting the disease outcome of *Salmonella*, the virulence potential and food source attribution of *Listeria monocytogenes*, as well as the abundance of *V. parahaemolyticus* (Tanui et al., 2022a; Ndraha et al., 2021; Karanth et al., 2022; Gmeiner et al., 2024). According to the models with good performance, the most influential predictors could also be retrieved, which shows great promise in managing and controlling food safety accurately. For example, Benefo et al. (2024a) adopted six different ML algorithms and identified the critical *Salmonella* stress response gene during poultry processing with high accuracy. Random forest (RF), as one of the most used ML algorithms in food safety, has been highlighted for its robust performance when the number of predictors is much larger than the number of observations, such as in WGS data (Biau and Scornet, 2016). Generally, the RF algorithm aggregates the prediction of several randomized decision trees through averaging, to obtain a final prediction/decision (Biau and Scornet, 2016). Thus, applying RF and alternative modeling efforts holds the potential to retrieve and reveal the information underlying bacterial behaviors from a genetic level via analyzing WGS data.

For this study, we aimed to perform a pangenomic analysis and apply RF to identify key genetic signatures of *V. parahaemolyticus* isolated from environmental, seafood, and clinical samples (i.e., potential differences in metabolism, virulence, and antibiotic resistance as a factor of source type). The findings from this study could help to (1) understand the adaptive response of *V. parahaemolyticus* as it transmits along the farm-to-table supply chain (environment-seafood-consumer) and (2) identify potential virulence factors and antibiotic resistance genes in *V. parahaemolyticus* that may have implications for consumer health and food safety.

## 2 Materials and methods

### 2.1 Sample collection

Genome assemblies of *V. parahaemolyticus* were collected from the National Center for Biotechnology Information (NCBI) Pathogen Detection database.^1^ A total of 6,227 assemblies consisting of environmental (*n* = 633), seafood (*n* = 2,284), and clinical (*n* = 3,310) isolates were downloaded and used in this study after checking the isolation type and isolation source manually for each assembly. Assemblies were subset for further analysis based on specific inclusion criteria for having corresponding metadata that indicated specific sample sources (i.e., environmental, seafood, and clinical), as described in Supplementary Table S1.

### 2.2 Bioinformatics analysis

The selected genome assemblies were processed with CheckM (v1.2.2) (Parks et al., 2015) for quality control, and those predicted to have greater than 97% completeness and less than 3% contamination (*n* = 176, 975, and 865 for environmental, seafood, and clinical isolates, respectively) were further processed (Blaustein et al., 2019). Annotation and pangenome construction of these high-quality assemblies were performed with Prokka (v1.14.6) and Panaroo (v1.3.4), sequentially (Seemann, 2014; Tonkin-Hill et al., 2020). Genes identified in the pangenome were categorized into three different sets based on their prevalence across all strains analyzed: core genes were present in over 95% of isolates, shell genes were found between 15 to 95% isolates, while cloud genes were defined as those with a prevalence less than 15% isolates (Livingstone et al., 2018). In addition to the comprehensive pangenome for all isolates, pangenomes for the subgroups of seafood and clinical isolates were constructed as well.

The nucleotide sequences of all gene clusters in the respective pangenomes were translated with Prodigal (v2.6.3) (Hyatt et al., 2010). Amino acid sequences were then screened for homology to proteins in the Database of Clusters of Orthologous Genes (COG), the Virulence Factor Database (VFDB) and the Comprehensive Antibiotic Resistance Database (CARD) using BLASTp (v2.14.1) (Camacho et al., 2009; Liu et al., 2022; Alcock et al., 2023) to identify the gene profiles with homology to features for metabolism, virulence and antibiotic resistance, respectively. During our preliminary analysis, different cutoff thresholds ranging from 99 to 50% (99, 98, 97, 96, 95, 90, 85, 80, 75, 70, 65, 60, 55, and 50%) were employed to query coverage and percent identity, as we aimed to get the threshold as high as possible while 50% is the common choice for BLASTp. The filtered genes with different thresholds were fed into RF models as the predictors. After comparing the performance of models (sensitivity, specificity, balanced accuracy, and Area Under the Receiver Operating Characteristics curve (AUROC)) using filtered genes with different cutoff values (Supplementary Tables S2–S7), the thresholds for both query coverage and percent identity were set as 90, 80, and 50% for metabolism, virulence, and antibiotic resistance models, respectively.

### 2.3 Machine learning

**Figure 1:**
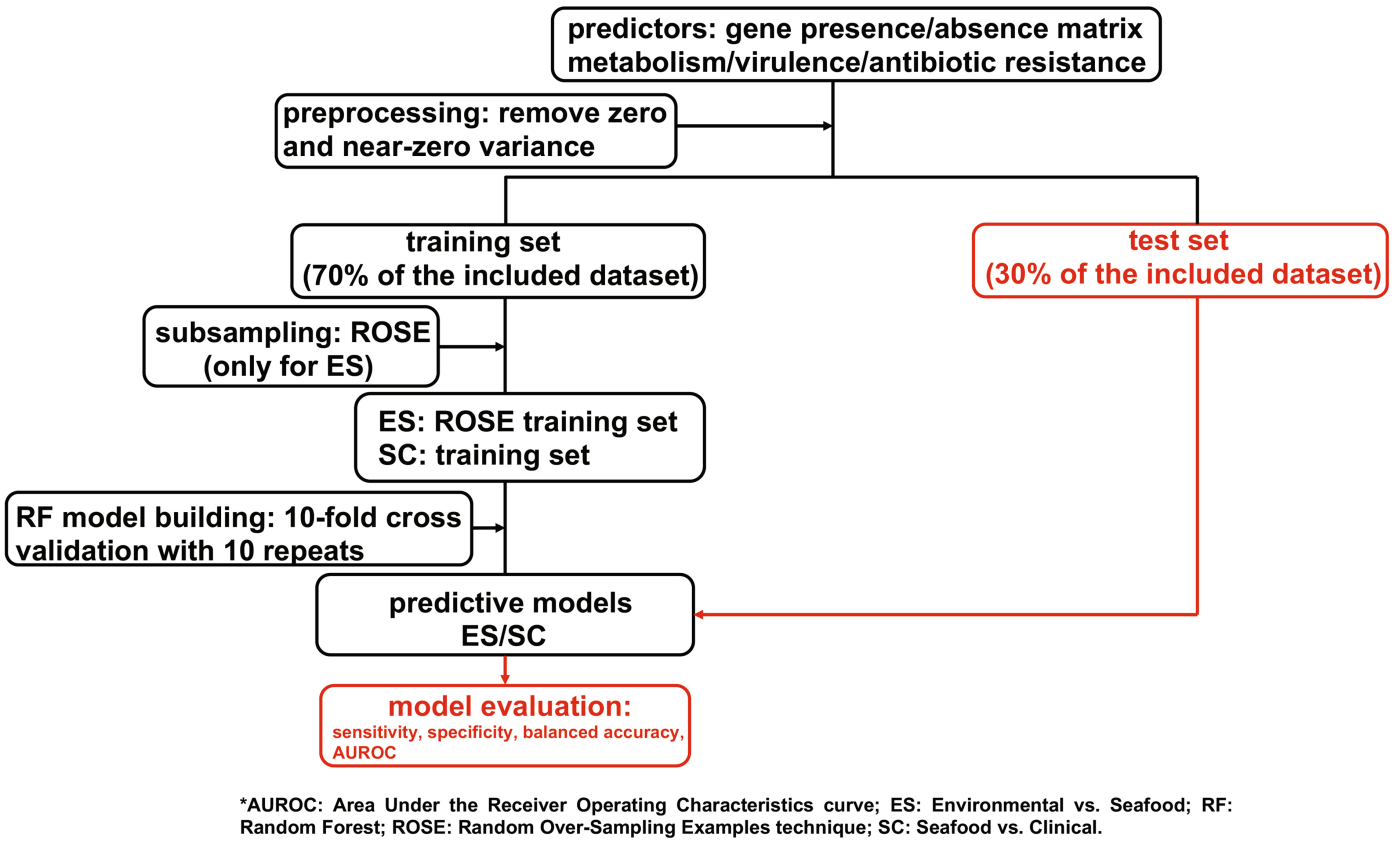
A simplified workflow for the approach used in this study.

Random forest was adopted to develop predictive models for isolation sources of *V. parahaemolyticus* (environmental vs. seafood (ES) and seafood vs. clinical (SC)). The presence and absence of genes related to metabolism, virulence, and antibiotic resistance were separately used as the predictors. The overview of the prediction strategy used in this study is simplified as a workflow and displayed in Figure 1. Further details regarding this approach are described in the following sections.

#### 2.3.1 Data preprocessing

The presence and absence of gene clusters (denoted by 1 and 0, respectively) with homology to each functional category (metabolism, virulence, and antibiotic resistance) were used as the input variables for the ML models. Predictors (gene clusters) possessing only one unique value (zero variance predictor) or a limited number of unique values (near-zero variance predictor) were removed as they could introduce unnecessary complexity to the model and lead to increased computational time without significantly increasing the accuracy of the model (Kuhn, 2019). Predictors with near-zero variance were detected by estimating frequency ratio (the frequency of the most prevalent value over the second most frequent value) and unique value percentage (the number of unique values to the total number of samples expressed as a percentage). For this study, a predictor with a frequency ratio greater than 19 and a unique value percentage less than 10% was considered as near-zero variance and, therefore, excluded from model building (Kuhn, 2019; Benefo et al., 2024a).

Class imbalance, which could result in potential bias in the model, was observed for ES (15.29% for the minority class (environmental isolates) and 84.71% for the majority class (seafood isolates)) while was not found in SC (47.01% for the minority class (clinical isolates) and 52.99% for the majority class (seafood isolates)). Upsampling, downsampling, random over-sampling examples (ROSE) technique, and Synthetic Minority Oversampling TEchnique were applied to attenuate the imbalance in the ES dataset during the preliminary analysis, and ROSE was selected for subsampling in the ES models due to having better predictive performance than the other methods. Through ROSE, the majority class is downsampled while new instances are generated via a smoothed-bootstrap approach for the minority class (Lunardon et al., 2014).

#### 2.3.2 Model building

Six classification models were developed using RF for both ES and SC datasets across each functional category: metabolism, virulence, and antibiotic resistance. For each model, the dataset was randomly split into a training set (70% of included data) and a test set (30% of included data), which were used for model building and model test and validation, respectively (Benefo et al., 2024b). Ten-fold cross-validation with 10 repeats was adopted to train the model as it helps to reduce the potential bias (Kohavi, 1995). Specifically, the training set was randomly partitioned into 10 subsets, and 10 models were built out of these 10 subsets. For each model/iteration, nine subsets were employed to train the model while the remaining set was kept aside to test and evaluate the model performance. The aforementioned procedures were repeated 10 times, resulting in an average performance for all models generated throughout the process (Kuhn, 2019). Randomized search was adopted to tune the hyperparameters and identify the optimal ones for each model. The test of the developed models was conducted using the hold-out test set (30% of included data), and a confusion matrix was generated according to model performance on the test set.

#### 2.3.3 Model evaluation

Sensitivity, specificity, balanced accuracy, and AUROC were used to evaluate the performance of the developed models. Sensitivity and specificity are commonly used metrics to evaluate the performance of classification models. Sensitivity is defined as the ratio of the correctly identified positives to all true positives, while specificity refers to the proportion of true negatives that are correctly predicted (Sidey-Gibbons and Sidey-Gibbons, 2019). Balanced accuracy, which is defined as the average of sensitivity and specificity, outperforms traditional accuracy when evaluating the performance of models with imbalanced data as it considers accuracies for both positive and negative classes (Thölke et al., 2023). Sensitivity, specificity, and balanced accuracy all range from 0 to 1; and the closer these values to 1, the better performance the model has. AUROC characterizes the classification (discrimination) ability of the model. Specifically, the value of AUROC varies from 0.5 to 1, with AUROC = 0.5 (baseline) linked to random classification while AUROC = 1 indicates a perfect classifier (D’Agostino et al., 2013). Moreover, the plots of AUROC were generated as well. In the AUROC graph, the false positive rate (1- specificity) of the model is the x-axis while the true positive rate (sensitivity) of the model is the y-axis. An AUROC curve which is close to the upper left corner of the graph is considered as the indicator of high AUROC value and therefore, good predictive ability of a model.

#### 2.3.4 Significant genes identification

The twenty most significant genes for each reliable model were identified and ranked based on their importance (note: only 17 genes were listed for the SC-antibiotic resistance model since it only had 17 genes as predictors). It was estimated by computing the difference in the prediction accuracies of the model caused by permuting the values of each predictor variable. The calculated difference between the two accuracies was averaged over all trees and normalized by the standard error. The more significantly permuting the value of a predictor impacts the accuracy, the more important that predictor (Kuhn, 2019). All the ML analyses were performed using the *caret* and *MLeval* package (Kuhn, 2019) in R (v. 4.1.1). The prevalence rate (the ratio of positive genomes to the total genomes) of the identified genes was calculated. The Proportion test was performed using the *prop.test* package in R (v. 4.1.1) to evaluate the homogeneity of proportions in different isolate sources. In addition, relevant information about the specific genes in COG, VFDB, and CARD that were homologous to the most important pangenome gene cluster predictors (e.g., homologous gene COG category) were retrieved from the respective databases.

### 2.4 Data visualization

A pie chart was generated for the pangenome for all isolates. AUROC curves and heatmaps were generated for the prevalence of the identified important genes via R (v. 4.1.1) using the *autoplot* and *pheatmap* packages, respectively.

## 3 Results

### 3.1 Pangenome characteristics

**Figure 2:**
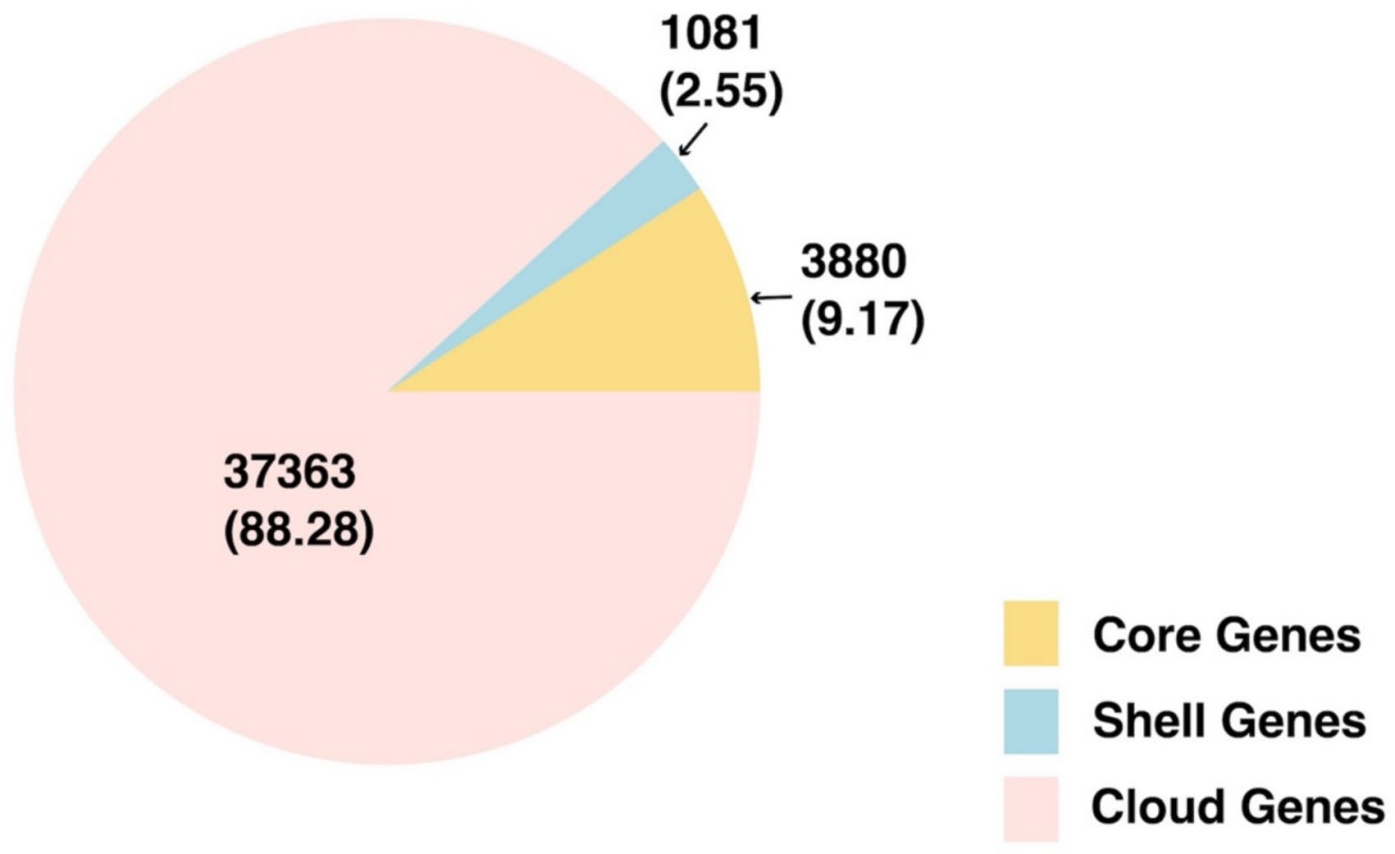
Pie chart of genes of the overall pangenome. Total genes (percent) are listed.

A total of 42,324 gene clusters were identified in the *V. parahaemolyticus* pangenome, with 4,608 ± 160 genes per genome (mean ± SD). Specifically, our pangenome identified 3,880 core genes, 1,081 shell genes, and 37,363 cloud genes. The pie chart demonstrating the distribution of total genes and respective percentages is shown in Figure 2.

### 3.2 Predictive models

Six ML classification models were built based on the presence and absence of genes with homology to metabolism, virulence, and antibiotic resistance for the ES and SC datasets. Based on the thresholds for query coverage and percentage of identity, 4,132, 273, and 160 genes were selected as inputs for the metabolism, virulence, and antibiotic resistance models, respectively. After removing zero and near-zero variance, 390, 23, 19, 380, 48, and 17 genes were used as the predictors for ES-metabolism, ES-virulence, ES-antibiotic resistance, SC-metabolism, SC-virulence, and SC-antibiotic resistance models, respectively.

The performance of all six models was measured using sensitivity, specificity, balanced accuracy, and AUROC (shown in Table 1). Generally, SC models provided better predictions compared to ES models, and models for metabolism surpassed those for virulence and antibiotic resistance.

**Table 1:** Model performance of the developed predictive models.

Models		Sensitivity	Specificity	Balanced accuracy	AUROC
ES	Metabolism	0.52	0.88	0.70	0.82
	Virulence	0.44	0.72	0.58	0.66
	Antibiotic resistance	0.52	0.76	0.64	0.70
SC	Metabolism	0.85	0.96	0.90	0.96
	Virulence	0.88	0.92	0.90	0.94
	Antibiotic resistance	0.73	0.87	0.80	0.87

ES, environmental vs. seafood; SC, seafood vs. clinical; AUROC, area under the receiver operating characteristics curve.

**Figure 3:**
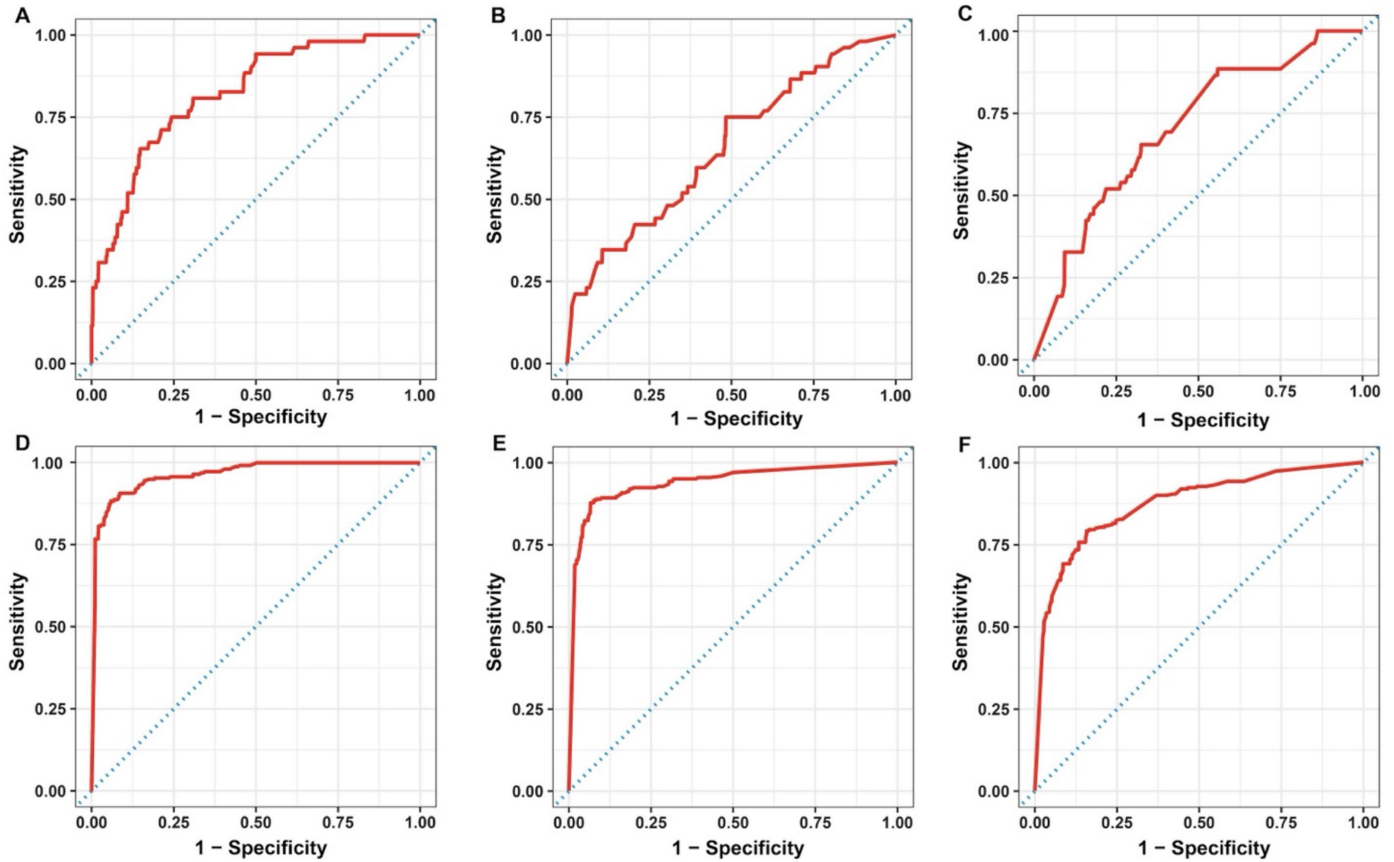
Area under the receiver operating characteristics curve for the developed RF models: ES-metabolism **(A)**, ES-virulence **(B)**, ES-antibiotic resistance **(C)**, SC-metabolism **(D)**, SC-virulence **(E)**, and SC-antibiotic resistance **(F)**. ES, environmental vs. seafood; SC, seafood vs. clinical.

Specifically, sensitivity, specificity, and balanced accuracy varied from 0.44 to 0.52, 0.72 to 0.88, and 0.58 to 0.70, respectively for ES models; while for SC models, the range for sensitivity, specificity, and balanced accuracy were 0.73 to 0.88, 0.87 to 0.96, and 0.80 to 0.90, respectively. On the other hand, all models, except for ES-virulence and ES-antibiotic resistance, resulted in an AUROC value greater than 0.80 (ranging from 0.82 to 0.96), and a model with an AUROC value above 0.80 is generally interpreted as a reliable model (Nahm, 2022). The plotted AUROC curves were shown in Figure 3 and the baseline, of which AUROC is equal to 0.5, was denoted as the dotted diagonal line in the graph.

Based on the overall consideration of four evaluation metrics, SC-metabolism, SC-virulence, and SC-antibiotic resistance models were considered as models which could provide robust prediction and were selected for further identification of significant genes.

### 3.3 Significant genes enriched by source type

**Figure 4:**
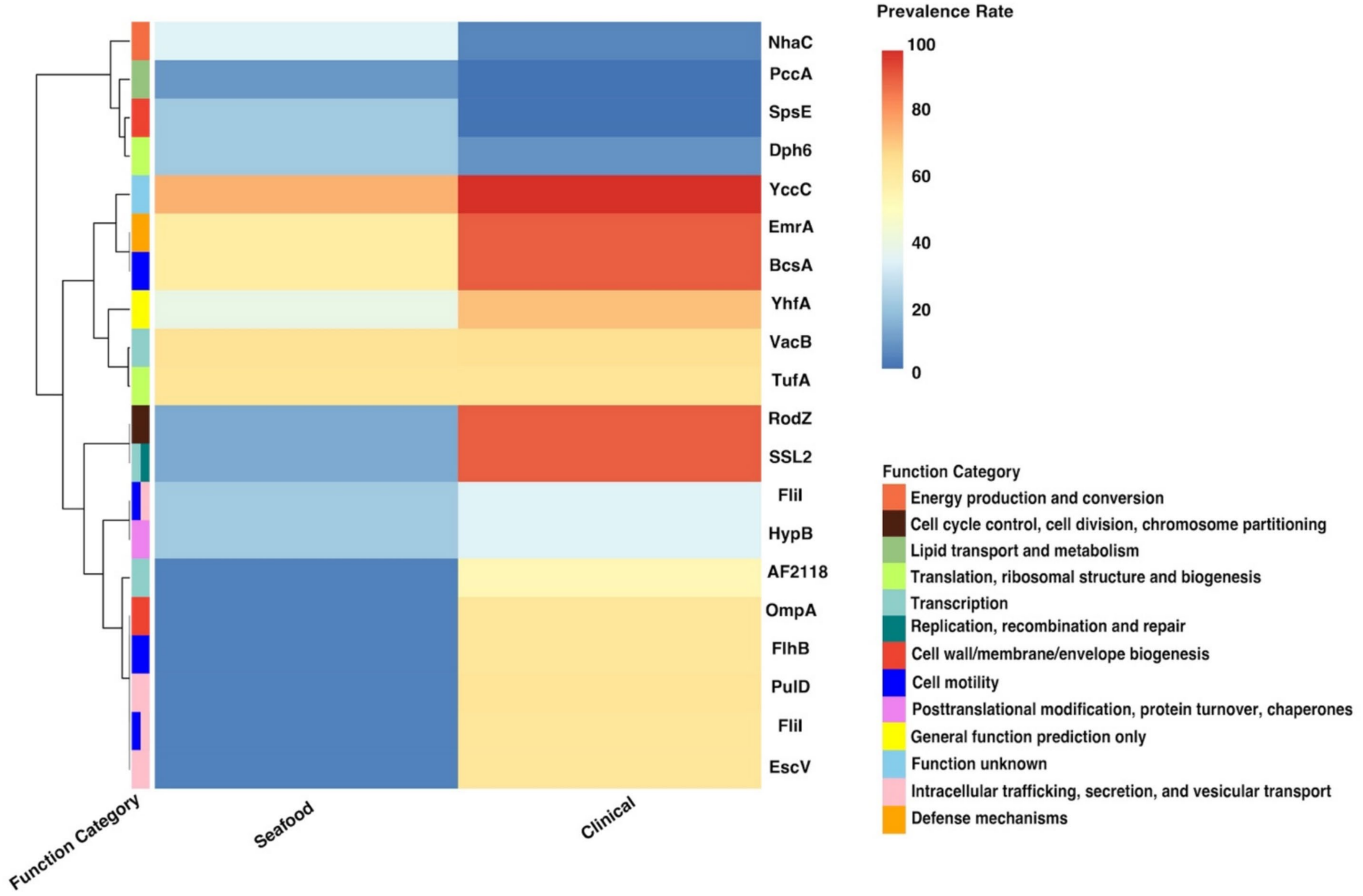
Heatmap for the prevalence of twenty important genes identified by the SC-metabolism model. SC, seafood vs. clinical.

**Figure 5:**
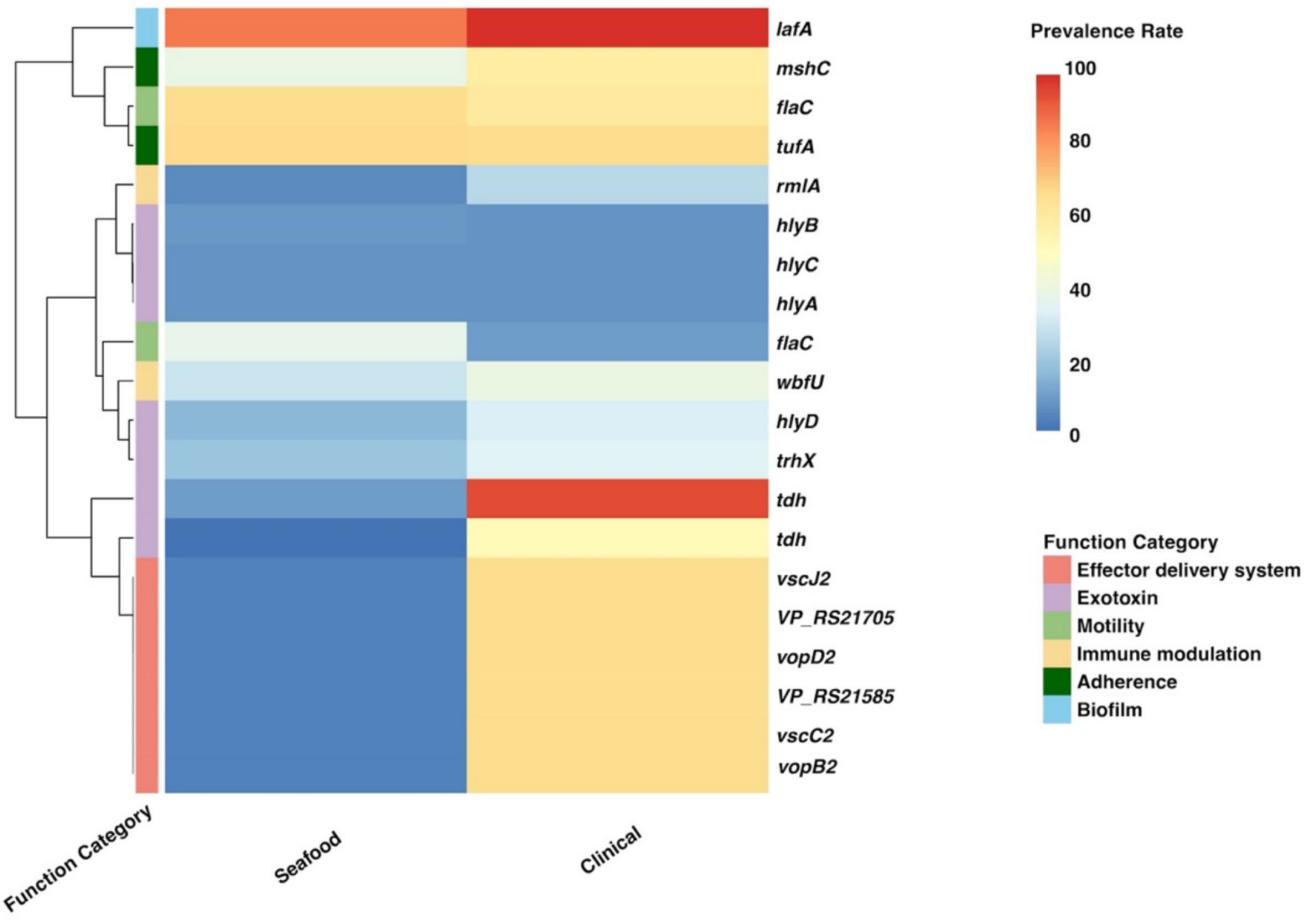
Heatmap for the prevalence of twenty important genes identified by the SC-virulence model. SC, seafood vs. clinical.

**Figure 6:**
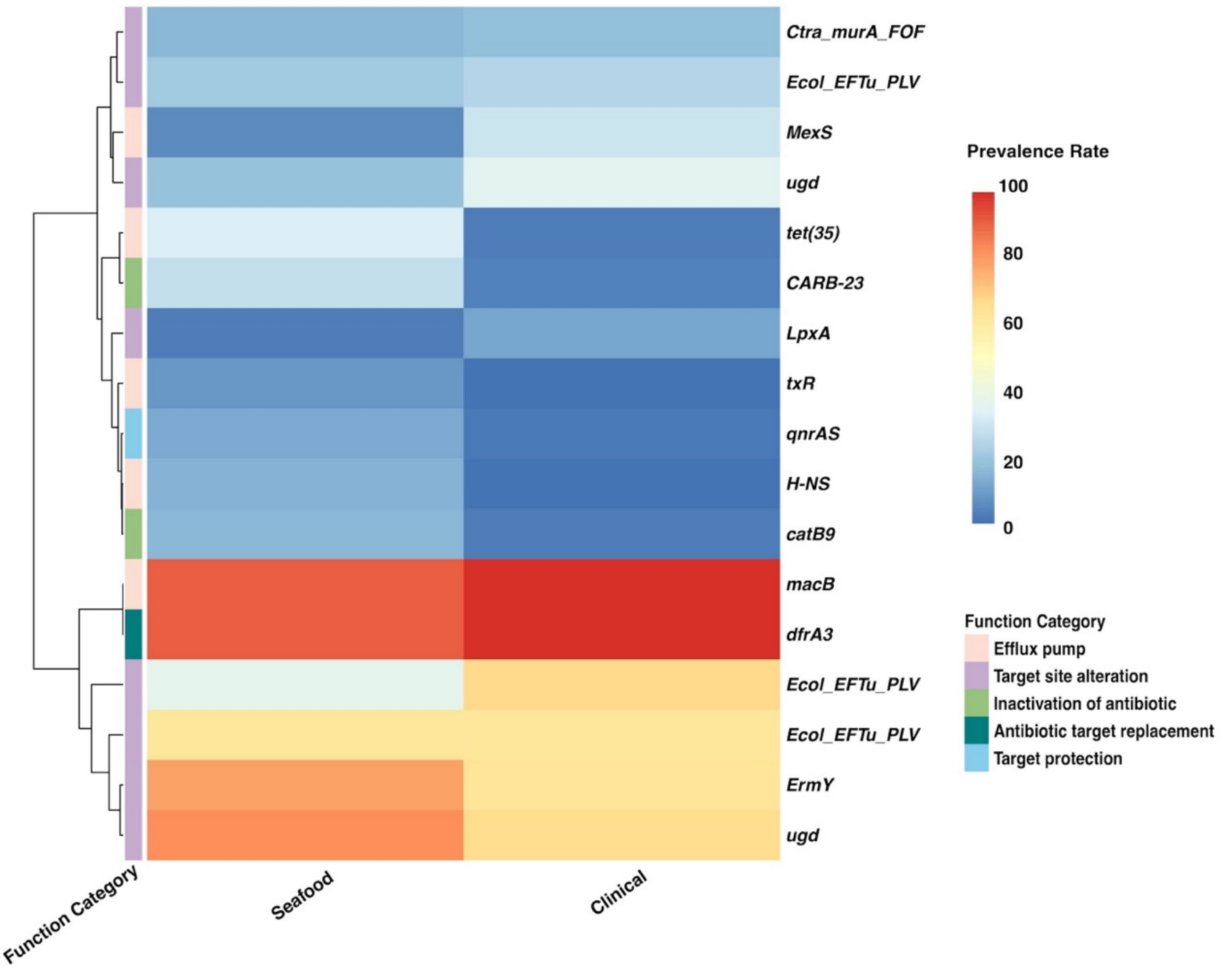
Heatmap for the prevalence of genes used as predictors in the SC-antibiotic resistance model. SC, seafood vs. clinical.

Twenty significant genes identified by the SC-metabolism and SC-virulence models, genes used as the predictors in the SC-antibiotic resistance model as well as the relevant information about their homologies in different databases and prevalence rates in the seafood and clinical groups were listed in Tables 2–4; and the related heatmaps were displayed as Figures 4–6.

**Table 2:** Twenty important genes identified by SC-metabolism model, information of their homologies from COG, and their prevalence rates.

Gene cluster from pangenome	COG annotation	COG symbol	COG name	COG functional category	Prevalence rate		*p* values
					Seafood	Clinical	
*group_1266*	Hypothetical protein VPA1391	RodZ	Cytoskeletal protein RodZ	D	12.10	87.98	< 0.001
*group_5540*	Hypothetical protein VPA1393	SSL2	Superfamily II DNA or RNA helicase	KL	12.10	87.98	< 0.001
*sctC_5~~~sctC_3*	Type III secretion system EscC protein	PulD	Type II secretory pathway component GspD/PulD (secretin)	U	3.69	60.69	< 0.001
*legI_2~~~legI*	N-acetylneuraminic acid synthetase	SpsE	Sialic acid synthase SpsE, contains C-terminal SAF domain	M	20.31	1.39	< 0.001
*yscN_2~~~atpB_1*	ATPase YscN	FliI	Flagellar biosynthesis/type III secretory pathway ATPase FliI	NU	3.69	60.58	< 0.001
*ssaV*	Type III secretion system EscV protein	EscV	Type III secretory pathway, component EscV	U	3.69	60.58	< 0.001
*group_268*	Outer membrane protein	OmpA	Outer membrane protein OmpA and related peptidoglycan-associated (lipo)proteins	M	3.69	60.46	< 0.001
*accA1_2~~~accA1*	acyl-CoA carboxylase alpha chain	PccA	Acetyl/propionyl-CoA carboxylase, alpha subunit	I	9.13	0.58	< 0.001
*yhfA_2~~~yhfA_1~~~yhfA_3*	Hypothetical protein VP1807	YhfA	Uncharacterized OsmC-related protein	R	36.92	69.60	< 0.001
*group_31591*	Hypothetical protein VP1134	NhaC	Na+/H+ antiporter NhaC/MleN	C	32.62	4.51	< 0.001
*hrcN*	Hypothetical protein	FliI	Flagellar biosynthesis/type III secretory pathway ATPase FliI	NU	20.10	32.37	< 0.001
*group_999*	Hypothetical protein VP1825	AF2118	Predicted transcriptional regulator, contains an XRE-type HTH domain (archaeal members contain CBS pair)	K	4.10	51.33	< 0.001
*ureG*	Urease accessory protein UreG	HypB	Hydrogenase/urease maturation factor HypB, Ni2 + −binding GTPase	O	20.41	32.37	< 0.001
*group_965*	Hypothetical protein VP2937	Dph6	Diphthamide synthase (EF-2-diphthine--ammonia ligase)	J	20.00	7.51	< 0.001
*rnr_1~~~rnr_2*	Virulence-associated protein VacB/Rnase R	VacB	Exoribonuclease R	K	62.36	62.77	0.892
*group_4703*	Hypothetical protein VPA0394	EmrA	Multidrug resistance efflux pump EmrA	V	57.74	87.51	< 0.001
*icaA*	Hypothetical protein VPA0393	BcsA	Glycosyltransferase, catalytic subunit of cellulose synthase and poly-beta-1,6-N-acetylglucosamine synthase	N	57.74	87.51	< 0.001
*flhB_3~~~yscU_2*	Type III secretion system EscU protein	FlhB	Flagellar biosynthesis protein FlhB	N	3.69	60.46	< 0.001
*aaeB~~~aaeB_1*	Hypothetical protein VP1358	YccC	Uncharacterized membrane protein YccC	S	72.51	95.95	< 0.001
*tufB~~~tuf~~~tuf1~~~tufA_2~~~tufA~~~tufA_1*	Elongation factor Tu	TufA	Translation elongation factor EF-Tu, a GTPase	J	61.33	60.92	0.895

COG, the Database of Clusters of Orthologous Genes. SC, seafood vs. clinical. C, Energy production and conversion; D, Cell cycle control, cell division, chromosome partitioning; I, Lipid transport and metabolism; J, Translation, ribosomal structure and biogenesis; K, Transcription; L, Replication, recombination and repair; M, Cell wall/membrane/envelope biogenesis; N, Cell motility; O, Posttranslational modification, protein turnover, chaperones; R, General function prediction only; S, Function unknown; U, Intracellular trafficking, secretion, and vesicular transport; V, Defense mechanisms.

**Table 3:** Twenty important genes identified by SC-virulence model, information of their homologies from VFDB, and their prevalence rates.

Gene cluster from pangenome	Name of the homologous gene in VFDB	VFDB gene product	VFDB functional category	Prevalence rate		*p* values
				Seafood	Clinical	
*tdh1_1~~~tdh3~~~tdh1~~~tdh2*	*tdh*	Thermostable direct hemolysin A	Exotoxin	9.13	86.24	< 0.001
*group_5343*	*vscJ2*	Type III secretion system protein VscJ2	Effector delivery system	3.69	60.81	< 0.001
*hlyD~~~hlyD_1~~~hlyD_2*	*hlyD*	Hemolysin D	Exotoxin	14.36	30.17	< 0.001
*hlyC_2*	*hlyC*	Hemolysin C	Exotoxin	7.28	7.40	0.995
*sctC_5~~~sctC_3*	*vscC2*	Type III secretion system protein VscC2	Effector delivery system	3.69	60.69	< 0.001
*hlyA~~~hlyA_2*	*hlyA*	Hemolysin A	Exotoxin	7.18	7.51	0.853
*group_9636*	*vopB2*	Type III secretion system translocator protein VopB2	Effector delivery system	3.69	60.69	< 0.001
*group_10785*	*mshC*	MSHA pilin protein MshC	Adherence	35.18	54.34	< 0.001
*epsL_1~~~epsL_2~~~pssY~~~epsL_3*	*wbfU*	Sugar transferase	Immune modulation	27.18	36.18	< 0.001
*group_6266*	*VP_RS21585*	Putative type III secretion system protein	Effector delivery system	3.69	60.69	< 0.001
*group_6750*	*vopD2*	Type III secretion system translocator protein VopD2	Effector delivery system	3.69	60.69	< 0.001
*flaD_4~~~flaD_2~~~flaD_5~~~flaD_1~~~flaD_3*	*flaC*	Flagellin	Motility	60.92	56.18	0.044
*tufB~~~tuf~~~tuf1~~~tufA_2~~~tufA~~~tufA_1*	*tufA*	Elongation factor Tu	Adherence	61.33	60.92	0.895
*hlyB~~~hlyB_2*	*hlyB*	Hemolysin B	Exotoxin	8.21	7.40	0.578
*group_10962*	*VP_RS21705*	Hypothetical protein	Effector delivery system	3.69	60.69	< 0.001
*tdh2~~~tdh2_1~~~tdh2_2*	*trhX*	TDH-related hemolysin	Exotoxin	17.95	32.14	< 0.001
*flaD_1~~~flaD_3*	*flaC*	Flagellin	Motility	34.05	9.60	< 0.001
*tdh3_2~~~tdh3~~~tdh1*	*tdh*	Thermostable direct hemolysin A	Exotoxin	0.62	48.09	< 0.001
*rffH_2~~~rffH*	*rmlA*	Glucose-1-phosphate thymidylyltransferase RfbA	Immune modulation	5.33	23.24	< 0.001
*hag*	*lafA*	Lateral flagellin LafA	Biofilm	78.46	90.75	< 0.001

SC, seafood vs. clinical. VFDB, the Virulence Factor Database.

**Table 4:** Genes used as predictors in SC-antibiotic resistance model, information of their homologies from CARD, and their prevalence rates.

Gene cluster from pangenome	Name of the homologous gene in CARD	Drug class	AMR gene family	Resistance mechanism	Prevalence rate		*p* values
					Seafood	Clinical	
*group_31591*	*tet(35)*	Tetracycline antibiotic	ATP-binding cassette (ABC) antibiotic efflux pump	Efflux pump	32.62	4.51	< 0.001
*tufA_1~~~tuf~~~tufA~~~tufB*	*Ecol_EFTu_PLV*	Elfamycin antibiotic	elfamycin resistant EF-Tu	Target site alteration	36.51	66.13	< 0.001
*group_5516*	*MexS*	Phenicol antibiotic, diaminopyrimidine antibiotic, fluoroquinolone antibiotic	resistance-nodulation-cell division (RND) antibiotic efflux pump	Efflux pump	6.77	29.60	< 0.001
*group_11708*	*ErmY*	Streptogramin antibiotic, lincosamide antibiotic, macrolide antibiotic	Erm 23S ribosomal RNA methyltransferase	Target site alteration	77.03	62.43	< 0.001
*group_8131*	*Ctra_murA_FOF*	Phosphonic acid antibiotic	antibiotic-resistant murA transferase	Target site alteration	16.92	18.15	0.539
*ugd~~~ugd_2~~~ugd_1*	*ugd*	Peptide antibiotic	pmr phosphoethanolamine transferase	Target site alteration	80.51	64.97	< 0.001
*macB_6~~~macB_4~~~macB_5~~~macB_3~~~macB_2*	*macB*	Macrolide antibiotic	ABC antibiotic efflux pump	Efflux pump	89.13	96.76	< 0.001
*ugd_1~~~ugd_2~~~ugd*	*ugd*	Peptide antibiotic	pmr phosphoethanolamine transferase	Target site alteration	19.69	35.26	< 0.001
*pse4*	*CARB-23*	Penam	CARB beta-lactamase	Inactivation of antibiotic	27.59	4.74	< 0.001
*tufB~~~tuf~~~tuf1~~~tufA_2~~~tufA~~~tufA_1*	*Ecol_EFTu_PLV*	Elfamycin antibiotic	elfamycin resistant EF-Tu	Target site alteration	61.33	60.92	0.895
*tufB~~~tufA~~~tufA_1~~~tufB_1~~~tuf1*	*Ecol_EFTu_PLV*	Elfamycin antibiotic	elfamycin resistant EF-Tu	Target site alteration	21.54	25.66	0.042
*dhfrIII*	*dfrA3*	Diaminopyrimidine antibiotic	trimethoprim resistant dihydrofolate reductase dfr	Antibiotic target replacement	89.13	97.11	< 0.001
*group_10971*	*LpxA*	Peptide antibiotic	Acinetobacter mutant Lpx gene conferring resistance to colistin	Target site alteration	4.10	12.83	< 0.001
*hns*	*H-NS*	Tetracycline antibiotic, penam, cephamycin, cephalosporin, fluoroquinolone antibiotic, macrolide antibiotic	RND antibiotic efflux pump, major facilitator superfamily (MFS) antibiotic efflux pump	Efflux pump	15.79	2.43	< 0.001
*cat_3~~~cat_2*	*catB9*	Phenicol antibiotic	chloramphenicol acetyltransferase (CAT)	Inactivation of antibiotic	16.31	3.82	< 0.001
*group_31739*	*qnrAS*	Fluoroquinolone antibiotic	quinolone resistance protein (qnr)	Target protection	13.95	3.58	< 0.001
*acoR_2~~~qseF~~~dctD_1*	*txR*	Tetracycline antibiotic	ABC antibiotic efflux pump	Efflux pump	9.64	1.85	< 0.001

AMR, antimicrobial resistance. CARD, the Comprehensive Antibiotic Resistance Database. SC, seafood vs. clinical.

As presented in the SC-metabolism model (shown in Table 2), the top 20 important genes were predicted as homologies to genes coding for proteins belonging to 13 different functional categories and intracellular trafficking, secretion, and vesicular transport, cell motility, as well as transcription were the most predominant categories. Most of the proportion of strains harboring the above genes (14 out of 20) were significantly greater in the clinical cohort than in the seafood group (displayed in Table 2; Figure 4).

According to the SC-virulence model (presented in Table 3), genes of great importance in characterizing the virulence profiles of seafood and clinical isolates belonged to six different functional categories and were primarily associated with exotoxin followed by effector delivery system. The proportion test revealed that the prevalence rates of 15 out of 20 important virulence genes differed significantly in seafood and clinical isolates. Among the genes with significantly different ubiquity, all of them were more encoded in clinical samples, other than ‘*flaD_1~~~flaD_3*’ (*flaC*) (Table 3; Figure 5).

Gene clusters used as predictors in our SC-antibiotic resistance model were predicted to resist 12 different drug classes including three multidrug classes (Table 4; Figure 6), among which genes conferring tetracycline resistance, elfamycin resistance, as well as multi-drug resistance (*tet(35)*, *Ecol_EFTu_PLV*, and *MexS*) were the top three important genes. The most common antibiotic resistance genes in the seafood cohort were *macB* (macrolide resistance, 89.13%), *dfrA3* (diaminopyrimidine resistance, 89.13%), and *ugd* (peptide resistance, 80.51%), while the most common antibiotic resistance genes in the clinical cohort were *dfrA3* (diaminopyrimidine resistance, 97.11%), *macB* (macrolide resistance, 96.76%), and *Ecol_EFTu_PLV* (elfamycin resistance; 66.13%). On the other hand, five different antibiotic resistance mechanisms were involved in differentiating the antibiotic resistance of seafood and clinical samples, and efflux pump, as well as target site alteration, were the two major categories.

## 4 Discussion

The overarching goal of this study was to use the differences in the presence and absence of genes among *V. parahaemolyticus* isolates as ML input to (i) develop classification models that differentiate *V. parahaemolyticus* isolates from environmental, seafood, and clinical samples, based on the accessory genes they carry that encode critical functions (metabolism, virulence, and antibiotic resistance) and (ii) identify the specific genes underlying the differences. Understanding potential mechanisms involved in transmission, pathogenicity, and antibiotic resistance of *V. parahaemolyticus* along the seafood supply chain could inform new strategies for food safety control and public health surveillance. To our knowledge, this is the initial attempt to adopt a bioinformatics workflow combined with ML to characterize differences in genetic diversity of *V. parahaemolyticus* strains across different isolation sources.

Our results showed that the three SC models could generate accurate predictions while the ES models did not perform as well. Therefore, only significant genes identified by SC models were analyzed and discussed. It is possible that compared with SC isolates, ES isolates were not that much different since these are all ‘commensal, possibly pathogenic’ strains recovered in monitoring while clinical strains are likely pathogens. However, limitations with the smaller sample size and data imbalance in the ES dataset may have affected the performance of ES models. In fact, significant biofilm formation was observed for *V. parahaemolyticus* in seafood compared with strains from the environment, implying the different lifestyles between environmental and seafood isolates (Rajkowski, 2009). Moreover, Feng et al. (2024) demonstrated that *V. parahaemolyticus* strains isolated from seawater and oyster were differently impacted by the same environmental parameters, indicating functional differences between certain environmental and seafood isolates as well. The inclusion of more environmental samples in the future should enable the model to capture and characterize the difference better.

In general, as shown in Table 2 and Figure 4, most of the top genes identified by our SC-metabolism model were more prevalent in clinical strains compared with seafood strains, indicating the more active metabolic activities occurring in clinical strains. This could be explained by the fact that the adaptative responses required to survive in the human body were more complicated than the ones associated with the seafood isolates due to the two distinguished conditions provided by the human body and seafood. When *V. parahaemolyticus* enters the human body, it could face various stresses such as thermal stress, acid stress, bile salts stress, and attack from the host cells, resulting in potential damage to different components of *V. parahaemolyticus* including cell membrane, DNA, and protein (Qadri et al., 2003; Pazhani et al., 2021). However, the stresses that seafood isolates may encounter are majorly associated with postharvest handling procedures such as cold stress caused by refrigeration storage and low salinity stress caused by washing (Huang and Wong, 2012; Tang et al., 2018). Thus, adaptive response of *Vibrio* along the processing and supply chain may become relevant for transmission and persistence that precedes consumption.

Specifically, the top two gene clusters (‘*group_1266*’ and ‘*group_5540*’), which were orthologous to cytoskeletal protein and superfamily II DNA or RNA helicase, were annotated as hypothetical proteins, pressing the need to study and reveal their functions and roles in the survival of *V. parahaemolyticus*. Intracellular trafficking, secretion, and vesicular, specifically, proteins associated with type III secretion system (T3SS), was one of the most predominant categories recognized by the SC-metabolism model and all the homologies (COG symbol: PulD, FliI, and EscV, ranked the third, fifth/eleventh, and sixth, respectively in the SC-metabolism model) belonging to this group were more prevalent in clinical isolates. Our findings were consistent with a previous study, in which the pangenome of *V. parahaemolyticus* was analyzed and significant enrichment of genes related to intracellular trafficking, secretion, and vesicular transport was observed for the clinical isolates (Pérez-Duque et al., 2021). This observation may be attributed to the fact that T3SS is a key virulence factor of *V. parahaemolyticus* (Li et al., 2019). Cell motility was the other most predominant functional category and four genes (COG symbol: FliI, FliI, BcsA, and FlhB, ranked the fifth, eleventh, seventeenth, and eighteenth, respectively, in the SC-metabolism model) out of the 20 important genes were recognized as the homologies to genes coding for proteins belonging to this category, particularly the orthologous cluster of flagellar biosynthesis. Similar to PulD and EscV, genes associated with flagellar biosynthesis were more frequently detected in clinical samples, highlighting the significance of flagellar in helping the transmission and survival of *V. parahaemolyticus* and possibly contributing to infection. It has been reported that the formation of biofilm, which is one of the important survival strategies of *V. parahaemolyticus*, is achieved with the aid of a dual flagellar system (Zhang Y. et al., 2023). On the other hand, the significantly high prevalence of four genes (COG symbol: SpsE, PccA, Nhac, and Dph6, ranked the fourth, eighth, tenth, and fourteenth, respectively) in seafood isolates could be explained by the response of the strain to the environmental pressure caused by the postharvest treatment of seafood. For example, *V. parahaemolyticus* has been reported to increase the expression of acetyl-CoA carboxylase (COG symbol: PccA) to synthesize unsaturated fatty acids and increase cell membrane fluidity to adapt to high hydrostatic pressure conditions, which has been commonly applied to inactivate the pathogen and extend the shelf life of seafood (Liang et al., 2022).

According to the SC-virulence model, exotoxin was the most predicted functional category (presented in Table 3). Specifically, two gene clusters were predicted to be two different copies of *tdh* and ranked first and eighteenth, respectively. On the other hand, one gene cluster from the pangenome was recognized as homology to *trhX* (also known as *trh*) and ranked sixteenth. These three gene clusters were significantly more prevalent in the clinical group, though none of them were present in *all* clinical isolates. Similar results have been found in previous studies, indicating the contribution of other factors to the pathogenicity of *V. parahaemolyticus* (Chao et al., 2010; Velazquez-Roman et al., 2012; Zha et al., 2023). Apart from homologies to *tdh* and *trhX*, homologies to four different alpha-hemolysin coding genes (*hlyD*, *hlyC*, *hlyA*, and *hlyB*) belonging to the exotoxin category have been identified as the top 20 influential predictors and ranked the third, fourth, sixth, and fourteenth, respectively. Interestingly, only *hlyD* was profoundly enriched in the clinical cohort compared with the seafood group while no significant difference was found regarding the prevalence rate of *hlyA*, *hlyB*, and *hlyC* in seafood and clinical isolates. In fact, the presence of *hlyA*, *hlyB*, *hlyC*, and *hlyD* in *V. parahaemolyticus* was only reported in a study investigating the pathogenesis of *V. parahaemolyticus* 353 isolated seafood in China (Zha et al., 2023). More studies are needed to reveal how these alpha-hemolysins contributed to the pathogenicity of *V. parahaemolyticus*, which could aid in explaining why their prevalence between seafood and clinical strains was similar but still critical to differentiate these two groups.

Moreover, it has been revealed that effector delivery system, T3SS, played an important role in differentiating nonpathogenic and pathogenic (seafood and clinical) groups. Based on our SC-virulence model, five genes related to T3SS (*vscJ2*, *vscC2*, *vopB2*, *VP_RS21585*, and *vopD2*) were identified as important genes and ranked the second, fifth, seventh, tenth, and eleventh, respectively. *V. parahaemolyticus* possesses two sets of T3SS: T3SS1 and T3SS2, which are responsible for cytotoxicity and enterotoxicity, respectively (Li et al., 2019). All the effector delivery system genes identified by the SC-virulence model were associated with T3SS2, which could be explained by the fact that T3SS1 is commonly found in both nonpathogenic and pathogenic isolates while T3SS2 is exclusive to pathogenic/clinical isolates (Matsuda et al., 2020). Generally, the proteins of T3SS could be categorized into four classes: structural proteins, translocators, effector proteins, and molecular chaperones (Li et al., 2019). In this study, we observed two genes predicted to encode structural proteins of T3SS2 (*vscJ2* and *vscC2*), which contribute to the formation of the physical structure of T3SS2, particularly the assemble of the inner membrane of both the basal body and export apparatus (Deng et al., 2017). Genes *vopB2* and *vopD2*, coding for the translocator protein of T3SS2, have been reported to be responsible for creating the pathway, pores in the membrane of host cells, through which effectors could be delivered into the host cells (Paria et al., 2021). It should be noted that two of the T3SS-related genes were hypothetical/putative proteins, which presses the need to perform further research specifically on these genes to unveil their characterizations and roles in contributing to the pathogenicity of *V. parahaemolyticus*.

Among all gene clusters identified as important by our SC-virulence model, the homology to *flaC* (ranked the seventeenth) was the only one that was more prevalent in the seafood group. It has been reported that FlaC, which is one of the flagellin subunits of the filament of *V. parahaemolyticus* flagellum coded by *flaC*, can activate the immune protection function of shellfish (Chen et al., 2019). We suspect that *flaC*-activated immune protection could result in changes in the texture or appearance of shellfish, causing consumers to perceive it as unsafe to eat. In contrast, shellfish contaminated with *V. parahaemolyticus* lacking *flaC* may not exhibit such changes, which makes people consider it as safe for consumption. Consequently, shellfish contaminated with *V. parahaemolyticus* lacking *flaC* is more likely to be eaten by consumers, which may explain the relatively lower prevalence of *flaC* in clinical isolates.

The gene *tet(35)*, which confers tetracycline resistance, was identified as the most important predictor in the SC-antibiotic resistance model with higher prevalence in seafood samples (shown in Table 4). Our results corresponded well with the frequently observed tetracycline resistance in seafood isolates worldwide (Elmahdi et al., 2016). *EFTu*, which confers to elfamycin resistance, ranked as the second among all the predictors in our SC-antibiotic resistance model. Several studies have described elfamycin resistance in pathogens obtained from various seafood and aquatic environments, which could be attributed to the usage of elfamycins as growth promoters for aquaculture (Behera et al., 2021; Liu et al., 2019; Zhang Q. et al., 2023). In addition, *MexS* (ranked the third), possessing multidrug resistance (phenicol antibiotic, diaminopyrimidine antibiotic, and fluoroquinolone antibiotic), were more predominantly found in the clinical group rather than the seafood cohort. The low prevalence rate of *MexS* in seafood samples (6.77%) observed in our study was consistent with previous research (Hanekamp and Bast, 2015; Obaidat et al., 2017; Lei et al., 2020; Kemp et al., 2021; Bondad-Reantaso et al., 2023).

Efflux pump and target set alteration were the most prevalent antibiotic resistance mechanisms associated with the predictors used by our SC-antibiotic resistance model (shown in Table 4). The presence of *tet(35)*, *MexS*, m*acB*, *H-NS*, and *txR* (ranked the first, third, seventh, fourteenth, and seventeenth, respectively), which are related to ATP-binding cassette (ABC), resistance-nodulation-cell division (RND), and major facilitator superfamily (MFS) antibiotic efflux pump, could be indicative of the essential roles of ABC, RND, and MFS efflux pumps in differentiating antibiotic resistance profiles of seafood and clinical isolates and similar insights have been gained from prior studies (Pérez-Acosta et al., 2018; Lloyd et al., 2019; Stephen et al., 2022). Though the target site alteration mechanisms of the listed important genes in *V. parahaemolyticus* (*Ecol_EFTu_PLV*, *ugd*, and *LpxA*) have not been extensively studied, the involvement of their related gene family in the antibiotic resistance have been demonstrated (Miele et al., 1994; Tracevska et al., 2002; Novović and Jovčić, 2023).

Additionally, the characterization of the individual pangenomes for the respective seafood and clinical isolates were summarized in Table 5. The total numbers of core genes and shell genes between the pangenomes of seafood and clinical isolates appeared similar, while the number of cloud genes for the seafood pangenome was about two-fold more than that for clinical pangenome, resulting in the drastic difference of the sizes of pangenome. The respective genes-per-genome by isolate source were consistent with this observation, indicating much greater genomic diversity of *V. parahaemolyticus* isolated from seafood samples. These differences may be attributed to the broader geographic distribution of isolation locations of the isolates from seafood samples compared to clinical samples. Horizontal gene transfer (HGT) of mobile genetic elements is commonly found in *V. parahaemolyticus* and has been proven that could greatly contribute to its genetic diversity (Xu et al., 2022). To be more specific, seafood isolates from diverse locations could obtain various genes through HGT, which explains the massive number of cloud genes in its pangenome.

**Table 5:** Summary table for the pangenomes of seafood and clinical isolates.

Pangenome	Core genes	Shell genes	Cloud genes	Total genes	Genes per genome (mean ± SD)
Seafood	3,886	877	32,543	37,306	4,629 ± 195
Clinical	4,017	1,025	14,325	19,367	4,580 ± 84

Although some models developed and used in this study could predict the isolation sources accurately and provide useful insights, certain limitations have been recognized. The limited availability of environmental isolates, which resulted in a severe class imbalance for our ES models, has constituted an obvious limitation and affected the robustness of the model greatly in this study. Though ROSE has been applied to overcome the bias caused by the imbalanced class and has significantly improved the model performance compared with models built based on data without ROSE, the obtained ES models were still not capable of providing accurate predictions. The scarcity of *V. parahaemolyticus* strains isolated from environmental samples has also been described in several other studies (Turner et al., 2013; Ronholm et al., 2016; Obaidat et al., 2017; Yan et al., 2020). Therefore, in the future, times of sampling events and detections of *V. parahaemolyticus* in environmental samples should be increased to aid in comprehending the population features of environmental strains more representatively. Moreover, as the genome assemblies were downloaded from the NCBI database, potential bias or batch effects among different studies (e.g., sequencing platform, sequencing depth, assembler) may have contributed to variations we observed.

A great number of tools with different mechanisms are available for each bioinformatic analysis step in this study and alternative tools may be resourceful to find additional differences correlated with the metadata. Therefore, the choice of method for each step could potentially impact our results. Although Prokka and Panaroo were used in this study, future work will explore other bioinformatics tools, such as PGAP, Roary, and PIRATE, to better understand how method selection may impact the downstream analysis. Additionally, the cutoff values for query coverage and percent identity were set based on the number of predictors, potentially impacting the performance of our random forest models. To enhance the robustness of our models, we systematically tested various BLASTp thresholds for query coverage and percent identity (as described in 2.2 Bioinformatics analysis), identifying the thresholds that yielded the most reliable predictions. In future studies, higher cutoff values should be applied when more datasets become available, as this may reduce noise associated with lower cutoff thresholds. Further research is needed to thoroughly assess how the choice of different bioinformatics tools influences downstream analysis and to develop a standardized and most optimal workflow for bioinformatics-ML studies.

Moreover, the prediction of gene function was greatly restrained by the size and accuracy of databases (COG, VFDB, and CARD) used for performing BLASTp analysis. It has been noticed that models for metabolism and virulence outperformed models for antibiotic resistance, which could be explained by the relatively limited predictors available for antibiotic resistance models, as the size of CARD is smaller than COG and VFDB. Expanding and updating respective gene function databases when new genes and functions are identified could contribute to overcoming this bias in the future. On the other hand, combining multiple databases might improve the performance of our models as well by providing a more comprehensive input. However, the lack of standardization and the methodological discrepancies between databases hinder the application of the database combination. Improved harmonization across databases and a thorough evaluation of the associated analysis method in the future could help address these challenges and make the combined database a feasible approach for enhancing model performance.

## 5 Conclusion

In this study, the application of machine learning was used to analyze pangenomes of *V. parahaemolyticus* to identify important genes associated with different isolation sources (environmental, seafood, and clinical). Our study highlights the crucial role of the type III secretion system in distinguishing metabolic and virulence accessory gene profiles of *Vibrio parahaemolyticus* seafood and clinical isolates. We also found that virulence-related genes encoding alpha-hemolysins were key in differentiating these groups. Among the top three most important predictors from our SC-antibiotic resistance model, gene conferring to tetracycline resistance was more prevalent in seafood isolates while genes confer to elfamycin, and multidrug (phenicol antibiotic, diaminopyrimidine antibiotic, fluoroquinolone antibiotic) resistance were greatly enriched in clinical isolates. These findings can help enhance risk management strategies along the seafood-to-consumer chain. However, the limited availability of environmental isolates significantly impacted the performance of our environmental-seafood model. Future research should focus on expanding sequencing databases for environmental samples and evaluating the impact of genomics workflow selection on analysis outcomes, providing a stronger scientific basis for selecting appropriate genomics tools.

## Data Availability

The original contributions presented in the study are included in the article/Supplementary material, further inquiries can be directed to the corresponding author.

## Footnotes

^1^https://www.ncbi.nlm.nih.gov/pathogens/, accessed on March 4, 2024.
