# Supplementary material for: Bioinformatics combined with machine learning unravels differences among environmental, seafood, and clinical isolates of Vibrio parahaemolyticus
Source: Front Microbiol. 2025 Mar 19;16:1549260. doi: 10.3389/fmicb.2025.1549260 (PMC11961994; doi:10.3389/fmicb.2025.1549260)
Supplement: Supplementary file 1 [file Data_Sheet_1.pdf]

# *Supplementary Material*

**Table S1** Inclusion criteria for *Vibrio parahaemolyticus* isolates.

| Category | Isolation Type | Isolation Source |
|---|---|---|
| **Environmental Isolates** | environmental/other;<br><empty> | seawater; water; environmental; estuarine water; shrimp pond; sediment; aquaculture pond water; environment; salt water; the shrimp farm in eastern Thailand; seawater from oyster bed; marine surface water; sea water; estuary; marine water; shrimp farm; continental water; freshwater; estuarine sediment; aquaculture pond; seagrass sediment; marine; the shrimp farm in north Vietnam |
| **Seafood Isolates** | environmental/other;<br><empty> | oyster; food; shrimp; marine oyster; seafood; raw shrimp; hepatopancreas; fish; freshwater food; clams; imported/domestic retail oyster; hepatopancreas (aquaculture farm); oysters; fresh seafood sold at markets and supermarkets; imported/domestic retail mussels; squid; hard clam; clam, seafood; frozen shrimp; eastern oyster from coastal estuary; tilapia; shrimp(sh); shrimps; imported/domestic retail clams; macerated stomach and hepatopancreas samples from shrimp; raw oyster; mussels; yellow croaker; shrimp hetopancreas; anadara tuberculosa; blunt-snout bream; dried shrimp; crab meat lump; crayfish; mollusc; blue mussel (Mytilus edulis); bream; gut of asian seabass; mollusk; shellfish; ahpnd infected litopenaeus vannamei; clam; crabs; diseased shrimp; imported shrimp; ahpnd infected Penaeus japonicus; aquaculture farm (hepatopancreas); aquatic product; arctic |

| | | |
|---|---|---|
| | | surf clams; bullhead; ems/ahpnd-diseased hepatopancreas; fresh fish |
| **Clinical Isolates** | clinical | patient; stool; clinical; feces; human; clinical sample from patient with gastroenteritis; clinic; anal swab; clinical sample; wound; stool sample; ear; human with diarrhea associated with seafood; clinical patient; clinical isolate; human with diarrhea associated with seafood; surveillance of cases of diarrhea patients; blister; blood; foot wound from a diabetic patient; human gut; oral swab; person1; person2; sample from a sick person; spinal wound; stool specimens |

**Table S2** ES-metabolism model performance with different cut-off thresholds for query coverage and percent identity.

| % threshold | Number of predictors | Sensitivity | Specificity | Balanced Accuracy | AUROC |
|---|---|---|---|---|---|
| **99** | 139 | 0.48 | 0.87 | 0.67 | 0.77 |
| **98** | 198 | 0.54 | 0.89 | 0.71 | 0.78 |
| **97** | 231 | 0.54 | 0.89 | 0.71 | 0.79 |
| **96** | 255 | 0.52 | 0.87 | 0.69 | 0.80 |
| **95** | 279 | 0.54 | 0.88 | 0.71 | 0.81 |
| **90** | 390 | 0.52 | 0.88 | 0.70 | 0.82 |
| **85** | 470 | 0.60 | 0.90 | 0.75 | 0.85 |
| **80** | 524 | 0.62 | 0.90 | 0.76 | 0.85 |
| **75** | 600 | 0.60 | 0.90 | 0.75 | 0.85 |
| **70** | 667 | 0.60 | 0.90 | 0.75 | 0.85 |
| **65** | 734 | 0.63 | 0.89 | 0.76 | 0.86 |
| **60** | 812 | 0.63 | 0.90 | 0.77 | 0.86 |
| **55** | 873 | 0.62 | 0.90 | 0.76 | 0.85 |
| **50** | 980 | 0.54 | 0.90 | 0.72 | 0.83 |

ES: Environmental vs. Seafood

AUROC: Area Under the Receiver Operating Characteristics Curve

**Table S3** SC-metabolism model performance with different cut-off thresholds for query coverage and percent identity.

| % threshold | Number of predictors | Sensitivity | Specificity | Balanced Accuracy | AUROC |
|---|---|---|---|---|---|
| **99** | 154 | 0.83 | 0.95 | 0.89 | 0.95 |
| **98** | 210 | 0.83 | 0.95 | 0.89 | 0.96 |
| **97** | 241 | 0.83 | 0.96 | 0.89 | 0.96 |
| **96** | 258 | 0.83 | 0.95 | 0.89 | 0.96 |
| **95** | 278 | 0.82 | 0.96 | 0.89 | 0.96 |
| **90** | 380 | 0.85 | 0.96 | 0.90 | 0.96 |
| **85** | 456 | 0.85 | 0.96 | 0.91 | 0.96 |
| **80** | 496 | 0.85 | 0.96 | 0.90 | 0.96 |
| **75** | 555 | 0.85 | 0.96 | 0.91 | 0.96 |
| **70** | 616 | 0.84 | 0.96 | 0.90 | 0.97 |
| **65** | 679 | 0.85 | 0.95 | 0.90 | 0.96 |
| **60** | 745 | 0.85 | 0.95 | 0.90 | 0.96 |
| **55** | 806 | 0.86 | 0.95 | 0.90 | 0.96 |
| **50** | 889 | 0.86 | 0.96 | 0.91 | 0.96 |

SC: Seafood vs. Clinical

AUROC: Area Under the Receiver Operating Characteristics Curve

**Table S4** ES-virulence model performance with different cut-off thresholds for query coverage and percent identity.

| % threshold | Number of predictors | Sensitivity | Specificity | Balanced Accuracy | AUROC |
|---|---|---|---|---|---|
| **99** | 4 | 0.81 | 0.45 | 0.63 | 0.67 |
| **98** | 5 | 0.92 | 0.32 | 0.62 | 0.71 |
| **97** | 6 | 0.77 | 0.53 | 0.65 | 0.70 |
| **96** | 6 | 0.77 | 0.53 | 0.65 | 0.70 |
| **95** | 6 | 0.77 | 0.53 | 0.65 | 0.70 |
| **90** | 9 | 0.75 | 0.47 | 0.61 | 0.70 |
| **85** | 17 | 0.54 | 0.71 | 0.62 | 0.68 |
| **80** | 23 | 0.44 | 0.72 | 0.58 | 0.66 |
| **75** | 41 | 0.54 | 0.70 | 0.62 | 0.67 |
| **70** | 54 | 0.56 | 0.71 | 0.63 | 0.70 |
| **65** | 70 | 0.52 | 0.73 | 0.63 | 0.73 |
| **60** | 87 | 0.56 | 0.75 | 0.66 | 0.75 |
| **55** | 104 | 0.54 | 0.73 | 0.64 | 0.75 |
| **50** | 135 | 0.54 | 0.77 | 0.65 | 0.76 |

ES: Environmental vs. Seafood

AUROC: Area Under the Receiver Operating Characteristics Curve

**Table S5** SC-virulence model performance with different cut-off thresholds for query coverage and percent identity.

| % threshold | Number of predictors | Sensitivity | Specificity | Balanced Accuracy | AUROC |
|---|---|---|---|---|---|
| **99** | 17 | 0.58 | 0.97 | 0.77 | 0.77 |
| **98** | 19 | 0.83 | 0.84 | 0.83 | 0.89 |
| **97** | 21 | 0.84 | 0.92 | 0.88 | 0.91 |
| **96** | 21 | 0.84 | 0.92 | 0.88 | 0.91 |
| **95** | 21 | 0.84 | 0.92 | 0.88 | 0.91 |
| **90** | 29 | 0.84 | 0.94 | 0.89 | 0.93 |
| **85** | 42 | 0.87 | 0.93 | 0.90 | 0.94 |
| **80** | 48 | 0.88 | 0.92 | 0.90 | 0.94 |
| **75** | 65 | 0.86 | 0.92 | 0.89 | 0.95 |
| **70** | 79 | 0.87 | 0.92 | 0.90 | 0.95 |
| **65** | 98 | 0.86 | 0.93 | 0.90 | 0.95 |
| **60** | 116 | 0.85 | 0.92 | 0.89 | 0.95 |
| **55** | 137 | 0.83 | 0.92 | 0.88 | 0.95 |
| **50** | 166 | 0.85 | 0.93 | 0.89 | 0.96 |

SC: Seafood vs. Clinical

AUROC: Area Under the Receiver Operating Characteristics Curve

**Table S6** ES-antibiotic resistance model performance with different cut-off thresholds for query coverage and percent identity.

| % threshold | Number of predictors | Sensitivity | Specificity | Balanced Accuracy | AUROC |
|---|---|---|---|---|---|
| **99** | 0 | N/A | | | |
| **98** | 0 | N/A | | | |
| **97** | 0 | N/A | | | |
| **96** | 0 | N/A | | | |
| **95** | 0 | N/A | | | |
| **90** | 0 | N/A | | | |
| **85** | 5 | 0.33 | 0.74 | 0.54 | 0.61 |
| **80** | 5 | 0.33 | 0.74 | 0.54 | 0.61 |
| **75** | 7 | 0.50 | 0.74 | 0.62 | 0.62 |
| **70** | 8 | 0.46 | 0.71 | 0.59 | 0.62 |
| **65** | 10 | 0.48 | 0.71 | 0.59 | 0.64 |
| **60** | 10 | 0.48 | 0.71 | 0.59 | 0.64 |
| **55** | 11 | 0.48 | 0.72 | 0.60 | 0.64 |
| **50** | 19 | 0.52 | 0.76 | 0.64 | 0.70 |

ES: Environmental vs. Seafood

AUROC: Area Under the Receiver Operating Characteristics Curve

N/A: Not Available

**Table S7** SC-antibiotic resistance model performance with different cut-off thresholds for query coverage and percent identity.

| % threshold | Number of predictors | Sensitivity | Specificity | Balanced Accuracy | AUROC |
|---|---|---|---|---|---|
| **99** | 0 | N/A | | | |
| **98** | 0 | N/A | | | |
| **97** | 0 | N/A | | | |
| **96** | 0 | N/A | | | |
| **95** | 0 | N/A | | | |
| **90** | 0 | N/A | | | |
| **85** | 3 | 0.93 | 0.30 | 0.62 | 0.67 |
| **80** | 3 | 0.93 | 0.30 | 0.62 | 0.67 |
| **75** | 5 | 0.58 | 0.77 | 0.68 | 0.73 |
| **70** | 6 | 0.58 | 0.79 | 0.69 | 0.73 |
| **65** | 8 | 0.70 | 0.74 | 0.72 | 0.76 |
| **60** | 8 | 0.70 | 0.74 | 0.72 | 0.76 |
| **55** | 9 | 0.70 | 0.74 | 0.72 | 0.80 |
| **50** | 17 | 0.73 | 0.87 | 0.80 | 0.87 |

SC: Seafood vs. Clinical

AUROC: Area Under the Receiver Operating Characteristics Curve

N/A: Not Available
